# Not only documentation

**DOI:** 10.4103/2229-3485.80372

**Published:** 2011

**Authors:** Viroj Wiwanitkit

**Affiliations:** *Wiwanitkit House, Bangkhae, Bangkok-10160, Thailand*

Sir,

I read the recent publication on “notes to file” with great interest. Hazra noted that “It is important that all of us stop and think before generating the next NTF, whether it is necessary to do so or can the documentation of this issue be done more appropriate elsewhere.”[1] I would like to share ideas on this work. First, I agree that good documentation system is very important and the standard method protocol for good documentation is required. However, as it can be seen, recording or not is dependent on the practitioner. The appropriateness of the note is discussed. The possible important thing in clinical research might be the ethics and good practice in research performing. The note can be of good evidence but we should not forget that some might generate notes and use them as fake evidences.
